# Medical Post-Traumatic Stress Disorder Symptoms in Children and Adolescents with Chronic Inflammatory Arthritis: Prevalence and Associated Factors

**DOI:** 10.3390/children12081004

**Published:** 2025-07-30

**Authors:** Leah Medrano, Brenda Bursch, Jennifer E. Weiss, Nicholas Jackson, Deborah McCurdy, Alice Hoftman

**Affiliations:** 1 David Geffen School of Medicine, Pediatric Rheumatology, University of California Los Angeles, Los Angeles, CA 90095, USA; dmccurdy@mednet.ucla.edu (D.M.);; 2 David Geffen School of Medicine, Psychiatry and Biobehavioral Sciences and Pediatrics, University of California Los Angeles, Los Angeles, CA 90095, USA; 3 Pediatric Rheumatology, Joseph M. Sanzari Children’s Hospital, Hackensack University Medical Center, Hackensack, NJ 07601, USA; 4 David Geffen School of Medicine, Medicine Statistics Core, University of California Los Angeles, Los Angeles, CA 90095, USA

**Keywords:** pediatric rheumatology, arthritis, pain, mental health

## Abstract

**Background:** Youth with chronic rheumatologic diseases undergo medical experiences that can lead to post-traumatic stress disorder (PTSD). Understudied in pediatric rheumatology, medical PTSD can be significantly distressing and impairing. **Objective:** This study explored the prevalence of medical PTSD symptoms in youth with chronic inflammatory arthritis and associated factors, including pain, disease activity, mental health history, and anxiety sensitivity. **Methods:** A cross-sectional study of 50 youth (ages 8–18) with juvenile idiopathic arthritis (JIA) and childhood-onset systemic lupus erythematous (cSLE) was conducted at a pediatric rheumatology clinic. Participants completed self-report measures assessing post-traumatic stress symptoms (CPSS-V), pain, anxiety sensitivity (CASI), pain-related self-efficacy (CSES), adverse childhood experiences (ACEs), and fibromyalgia symptoms (PSAT). Clinical data included diagnoses, disease activity, treatment history, and demographics. **Results:** Forty percent had trauma symptoms in the moderate or more severe range. The 14% likely meeting criteria for probable medical PTSD were older (median 17 vs. 15 years, *p* = 0.005), had higher pain scores (median 4 vs. 3, *p* = 0.008), more ACEs (median 3 vs. 1, *p* = 0.005), higher anxiety sensitivity scores (median 39 vs. 29, *p* = 0.008), and higher JIA disease activity scores (median cJADAS-10 11.5 vs. 7.5, *p* = 0.032). They were also more likely to report a history of depression (71 vs. 23%, *p* = 0.020). No associations were found with hospitalization or injected/IV medication use. **Conclusions:** Medical trauma symptoms are prevalent in youth with chronic inflammatory arthritis. Probable PTSD was associated with pain and psychological distress. These findings support the need for trauma-informed care in pediatric rheumatology.

## 1. Introduction

Children and adolescents with more exposure to adverse childhood experiences (ACEs) have been shown to be at increased risk of chronic pain and functional disability [1,2]. Inversely, children with chronic pain are more likely to have had early life stressors and signs of both psychological and physical distress [3]. Studies have shown that both psychological and biological maladaptive responses to stressful events may be implicated in the development of chronic pain in youth [4].

For some youth, these stressors come in the form of traumatic medical experiences. Medical trauma is defined as “a set of psychological and physiological responses of children and their families to pain, injury, serious illness, medical procedures, and invasive or frightening treatment experiences” according to the National Child Traumatic Stress Network [5]. Research suggests that pain can be a source of traumatic stress as well as a potential negative outcome of traumatic experiences [6].

Medical trauma symptoms, or post-traumatic stress symptoms (PTSSs) related to medical experiences, have been described in children with chronic diseases, with some having clinically significant symptoms consistent with post-traumatic stress disorder (PTSD). Reported rates of PTSSs in medically ill children have ranged from 25 to 38%, with a 2020 meta-analysis of children with chronic diseases finding that 11.5% met PTSD criteria [7,8,9]. This analysis found that PTSSs were positively associated with higher illness severity and intensity of treatment and/or duration and decreased treatment adherence. A sample of healthcare providers found that 99% felt that medical trauma symptoms had negative effects on health outcomes in pediatric chronic illness [10]. However, the same study revealed that few providers participated in trauma-informed care, such as screening for past traumatic experiences (within and outside the medical context), learning about the impact of those traumatic experiences on the patient, and co-creating a treatment approach designed to avoid future inadvertent trauma.

Anxiety sensitivity refers to the fear of physical sensations (e.g., rapid heartbeat, dizziness, growling stomach or other unusual feeling) and the belief that these sensations may lead to harmful or negative outcomes. People with high anxiety sensitivity may interpret physical symptoms as a sign of a catastrophic medical problem. The fear and hypervigilance associated with anxiety sensitivity contribute to the intensity of trauma-related and pain-related distress [11,12,13].

Surprisingly, there has been limited data on medical trauma in chronic pediatric rheumatologic diseases. Juvenile idiopathic arthritis (JIA) and childhood-onset systemic lupus erythematosus (cSLE) are two rheumatologic diseases that can be characterized by chronic pain from inflammatory arthritis. Throughout their disease course, children with JIA and cSLE may encounter potentially traumatic medical experiences. Most have frequent office visits and blood draws for disease or medication monitoring, and some will be hospitalized. Many require treatment via potentially painful recurrent infusions and subcutaneous or intra-articular injections. In a 2012 study from the Childhood Arthritis and Rheumatology Research Alliance (CARRA) Registry, 46% of patients with JIA had been treated with intra-articular glucocorticoids and 45% with a biologic disease-modifying antirheumatic drug (DMARD) [14]. Analysis of a national database by Knight et al. (2014) showed a hospitalization rate of 8.6 per 100,000 children with SLE [15]. Despite the frequent exposure to potentially painful and distressing experiences from their rheumatic disease and medical care, little is known about medical trauma in this population.

This study aims to determine the prevalence of medical trauma and explore its associated factors in a small sample of children with chronic inflammatory arthritis. We hypothesize that medical trauma is underrecognized in this population and that it is associated with increased pain. We also anticipate medical trauma to be associated with the use of injected or intravenous medications and hospitalization.

## 2. Materials and Methods

### 2.1. Study Design and Participants

This is a cross-sectional exploratory study of children and adolescents aged 8 to 18 years with a diagnosis of JIA according to the International League of Associations for Rheumatology (ILAR) classification criteria, or cSLE with a history of inflammatory arthritis according to the 2019 European League Against Rheumatism (EULAR) and American College of Rheumatology (ACR) classification criteria. This age range was selected based on the validated ages for the scales described below in the “Questionnaire measures” section. Participants were recruited from a pediatric rheumatology clinic at a single academic center. Potential participants were identified and approached by a pediatric rheumatology fellow or faculty during a clinic visit between October 2022 and May 2023. Children and adolescents with potential confounders for joint pain, including injury to an affected arthritis joint within three months or a diagnosis of malignancy, were excluded from this study. Participants, and in some cases their guardian or parent, were asked to complete the tools and questionnaires described below at the time of their clinic visit. Consent was obtained from participants or their parent/guardian for those below 18 years. Assent was obtained from adolescents aged 13–17. This study was approved by the University of California, Los Angeles institutional review board.

### 2.2. Demographic and Clinical Characteristics

Demographic data were obtained from electronic medical record review, including age, self-reported sex, rheumatologic diagnosis, and medication history. Further demographic data, which included self-reported race, ethnicity, annual household income, and history of physician’s diagnosis of anxiety, depression, fibromyalgia, or PTSD, were collected via a multiple-choice form. Participants aged 18 years self-completed the demographics form, while parents/guardians completed the form for participants under the age of 18. Disease activity data were collected from medical records and included the 10-joint clinical Juvenile Arthritis Disease Activity Score (cJADAS-10) and Systemic Lupus Erythematosus Disease Activity Index 2000 (SLEDAI-2K) calculated at the time of enrollment. Both the cJADAS-10 and SLEDAI-2K have cut-off scores to differentiate clinically active from inactive disease [16,17]. Lab values included erythrocyte sedimentation rate (ESR) and c-reactive protein (CRP) if available within three months of enrollment.

### 2.3. Questionnaire Measures

CPSS-V: The Child PTSD Symptom Scale for DSM-V (CPSS-V) is a validated measure of PTSD in youth aged 8–18 [18]. The self-report version of CPSS-V prompts the respondent to rate 20 PTSD symptoms on a 5-point scale with this written form. Scores range from 0 to 80, and a cut-off score of 31 is used to identify probable PTSD diagnosis. Scores can be categorized by severity, including minimal, mild, moderate, severe, and very severe. The CPSS-V and its adult counterpart, the PTSD Checklist for DSM-5 (PCL-5), have been used in studies of medical trauma in other chronic diseases [19,20]. The original CPSS-V asks youth to identify their own traumatic experiences. However, an adapted version of the scale was used in this study, which asked participants to consider their experiences specific to arthritis with the following prompt: “Think about what it has been like to have arthritis. Some kids with arthritis have to do things that might be scary or upsetting, like going to the doctor a lot, staying in the hospital, taking medications by mouth or with needles, and getting their blood drawn. These questions ask about how you feel about your arthritis.” A similar cut-off score of 31 was used to identify probable medical PTSD. Participants who scored above the cut-off score were contacted and offered a referral to a social worker or mental health provider. All participants self-completed the CPSS-V.

CSES: The Child Self-Efficacy Scale (CSES) is a self-report tool used to measure self-efficacy related to normal functioning of a child when in pain. This is a 7-item measure prompting youth aged 8 or older to rate their confidence in performing certain activities when in pain on a 5-point scale, with total scores ranging from 7 to 35. Lower scores indicate higher self-efficacy when in pain. The scale has shown strong evidence for reliability and validity and has correlations with functioning and somatic symptoms [21]. All participants self-completed the CSES.

CASI: Anxiety sensitivity, or the belief that anxiety-related bodily symptoms have negative consequences, was measured with the Childhood Anxiety Sensitivity Index (CASI). The CASI is an 18-item self-report scale for children and adolescents aged 6 or older. Youth rate how aversively they view anxiety symptoms [22]. Scores range from 3 to 54, with higher scores indicating increased anxiety sensitivity. All participants self-completed the CASI.

ACEs: Adverse childhood experiences (ACEs) were reported with the Pediatric ACEs and Related Life-events Screener (PEARLS) part 1, developed by the Bay Area Research Consortium on Toxic Stress and Health (BARC) [23]. PEARLS part 1 includes 10 questions, and scores ranging from 0 to 10 represent the total number of adverse experience categories reported. The present study used de-identified versions of the parent/caregiver report for participants under the age of 18 years and self-report for participants aged 18 years.

PSAT: The Pain and Symptom Assessment Tool (PSAT), a recently developed self-report measure based on the adult 2010 ACR criteria for fibromyalgia, was used to assess the presence of comorbid juvenile fibromyalgia (JFM) [24]. The PSAT consists of the Widespread Pain Index (WPI) and the Symptom Severity (SS) scale and contains 72 items. Scores with WPI ≥ 7 and SS ≥ 5 or WPI 3–6 and SS ≥ 9 were considered consistent with JFM. Validation studies are ongoing. All participants self-completed the PSAT, and study staff were available at the time of administration to answer any clarifying questions.

Pain numerical rating scale: Participants verbally reported their average arthritis pain level over the past one week, with an 11-point scale ranging from 0 to 10.

### 2.4. Statistical Analysis

Summary statistics for this exploratory study are presented as median with 25th and 75th percentiles or N with relative frequency. Wilcoxon–Mann–Whitney rank sum and exact tests were used to assess differences between those with and without probable medical PTSD (CPSS-V score < 31 vs. ≥31). All analyses were conducted in R version 4.2.0 [25].

## 3. Results

### 3.1. Overall Study Population

Fifty children and adolescents enrolled in this study, with an average age at enrollment of 15 years and average time since diagnosis of their rheumatologic disease 5.4 years. Thus, 33 (66%) of the participants self-identified as female, and 29 (58%) self-identified their race as white. Eighteen (36%) self-identified as Hispanic or Latino/a. Forty-two (84%) were diagnosed with JIA of any subtype and eight (16%) with cSLE with a history of arthritis. The subtypes of JIA and demographic characteristics of the study participants are shown in Table 1. Diagnosis of depression was self-reported in 15 (30%) and anxiety in 21 (42%) overall.

### 3.2. Trauma Scores and Participant Characteristics

**Figure 1:**
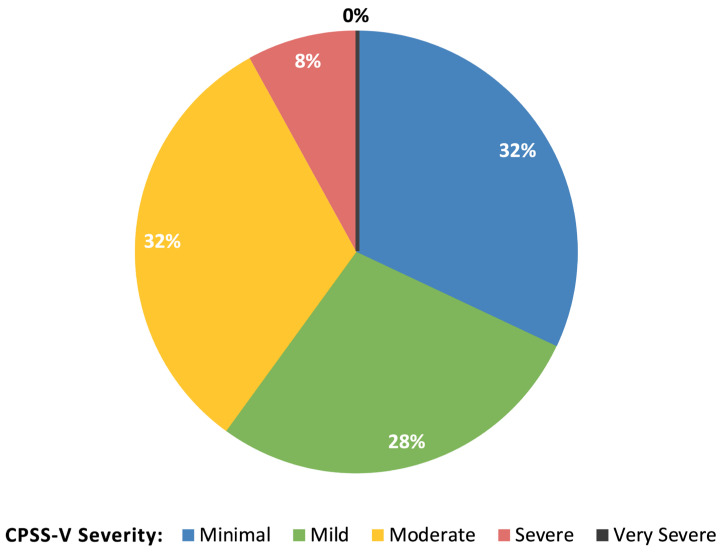
Percentage of children and adolescents with inflammatory arthritis with CPSS-V scores in each symptom severity category. Abbreviations: CPSS-V = Child PTSD Symptom Scale for DSM-V.

The overall median CPSS-V score was 14, and 20 (40%) children and adolescents had scores of moderate or greater severity. The percentage of participants scoring within each CPSS-V severity category is depicted in Figure 1. Seven (14%) adolescents scored 31 or above, within the probable medical PTSD range, including six with JIA and one with cSLE. There were similar rates of probable medical PTSD among participants with JIA (14%) and cSLE (13%). Participant characteristics stratified by CPSS-V scores above or below the probable medical PTSD range are described in Table 1. Those with probable medical PTSD were teens between ages 13 and 18 and were significantly older (median 17 vs. 15 years, *p* = 0.027), though with similar time since diagnosis. Descriptively, more participants with probable medical PTSD self-reported as white compared to their counterparts, but there were no significant differences in race and ethnicity between the two groups overall. Annual household income was similar above and below the medical PTSD cut-off score. Those with probable medical PTSD were more likely to report a history of depression (71 vs. 23%, *p* = 0.020). A similar trend was observed for a reported history of anxiety that did not meet significance.

### 3.3. Treatment History and Disease Activity

Treatment history and disease activity data are summarized in Table 2, broken down by CPSS-V scores above and below the cut-off for probable medical PTSD. Thirty-five (68%) participants had a history of intravenous, intraarticular, or subcutaneous medication use, and fifteen (30%) had been hospitalized for their rheumatic disease. A higher proportion of cSLE patients had a hospitalization history compared to those with JIA (87.5% vs. 19.0%, *p* = 0.000431). Probable medical PTSD status was not associated with a history of hospitalization or intravenous or subcutaneous medication use. The average cJADAS-10 score was 7.38, consistent with moderate disease activity. The cJADAS-10 average active joint count was 2.5, physician global assessment score 2.5, and patient/guardian global assessment score 3.5. The average SLEDAI-2K score was 7.88, indicating clinically active disease. cJADAs-10 scores were significantly higher in the probable medical PTSD group compared to their counterparts (median 11.5 vs. 7.5, *p* = 0.032) (Table 2). There were no differences in the available ESR and CRP values in those above the medical PTSD cut-off compared to those below. However, there were 11 and 14 unavailable ESR and CRP values, respectively.

### 3.4. Other Questionnaire Scores

For all participants, the median number of ACEs was two. Significantly more ACEs were reported in the children and adolescents scoring in the medical PTSD range than those below (median 3 vs. 1, *p* = 0.005). Participants reported a median pain score of 4 (range 0 to 10). Those with CPSS-V scores within the medical PTSD range had significantly higher pain scores compared to their counterparts (median 4 vs. 3, *p* = 0.043). The median CSES score was 18.21, with a trend toward higher scores in the medical PTSD group. Nine (18%) participants met the criteria for JFM on the PSAT. Relatively more children with CPSS-V scores in the medical PTSD range met the criteria for JFM compared to their counterparts (43% vs. 14%, *p* = 0.100), but this did not reach statistical significance. The isolated SS scores from the PSAT were significantly higher in the probable medical PTSD group (median 8 vs. 5, *p* = 0.039). The median CASI score for all participants was 29.5. CASI scores were significantly higher for those with CPSS-V scores within the probable medical PTSD range compared to those below (median 39 vs. 29, *p* = 0.008). The above questionnaire scores are presented in Table 3.

## 4. Discussion

In this exploratory study of children and adolescents with chronic inflammatory arthritis secondary to JIA or cSLE, medical trauma symptoms were reported by many participants. We found that 14% of participants reported sufficient symptoms to be categorized as having probable medical PTSD, and 40% reported medical trauma symptoms of at least moderate severity. The rates of PTSD seen in our study are similar to those reported in other children with chronic illnesses ranging from 10 to 20% [26].

Contrary to our prediction, a history of exposure to the potentially painful experiences of intravenous or injected medications and hospitalization, as collected from the medical record, was not associated with increased medical trauma symptoms. This differs from the findings of Pinquart (2020), who showed that higher post-traumatic stress symptoms were related to treatment intensity in pediatric patients with chronic illness [7]. The majority of participants in the present study had never been hospitalized for their rheumatologic disease, which we suspect impacted the ability to observe associations. However, it could be that those with high CPSS-V scores in our study found other medical experiences to be more traumatic than those we measured. Medical trauma was associated with increased pain scores overall, which likely reflects illness-related pain. This finding suggests that illness-related pain may be more distressing than one’s history of hospitalization or administration of medication via potentially painful routes. This hypothesis requires further study.

A higher percentage of patients with probable medical PTSD met the criteria for JFM, a condition of chronic widespread pain, though this did not reach statistical significance. This trend is consistent with prior descriptions of comorbid JFM and PTSD [27,28]. With a larger sample size, we suspect that those with JFM who have higher pain levels would have been found to have a greater risk for PTSD.

Overall, disease activity scores for both JIA and cSLE participants in the present study were relatively high. There was a positive association between higher JIA disease activity and medical PTSD. There was no significant correlation found between cSLE disease activity and medical PTSD, likely due to low power as only one participant qualified in this category. Of note, the cJADAS-10 includes a patient/parent global assessment, while the SLEDAI-2K does not. However, differences between the physician and patient/parent global assessment scores in the cJADAS-10 did not account for the higher JIA disease activity scores seen in the probable medical PTSD subgroup.

Previous studies have shown associations between PTSD and rheumatic diseases. The connections between PTSD and SLE have been well reported in the literature, with a review of the links published by Goldschen et al. (2023) [29]. A large study assessing PTSD and incident SLE cases in Medicaid recipients found that patients with preexisting PTSD had two-times the odds of developing SLE compared to those without PTSD [30]. Similar associations have been made with ACEs and other stressors, and we found that more ACEs were reported by the probable medical PTSD subgroup. Rubinstein et al. (2020) found more ACEs in children with arthritis compared to their healthy counterparts [31]. Stressors, including trauma and ACEs, in adults with SLE have been associated with flares and disease activity in studies by Patterson et al. and Katz et al. [32,33].

We observed a trend toward higher self-efficacy scores, indicating decreased self-efficacy, in the probable medical PTSD subgroup compared to their counterparts. Decreased self-efficacy has been linked to pain and disability in people with arthritis [34]. Vuorimaa et al. (2008) demonstrated that JIA patients with higher self-efficacy had less pain, anxiety, and depression [35]. Though our study did not directly assess for inter-associations between these factors, the children with probable medical PTSD similarly tended to have poorer self-efficacy, more pain, increased anxiety sensitivity, and reported history of depression.

In this study, we found that youth with probable medical PTSD were more likely to self-report a history of depression and have higher anxiety sensitivity. Pre-existing mental health disorders are known predictors of PTSSs in children [12,36]. Although reporting a known history of anxiety did not reach significance, an association was observed with our measure of anxiety related to somatic symptoms and probable PTSD. Anxiety sensitivity has been found to intensify the fear of pain and has been associated with disability related to pain, as well as decreased health-related quality of life [37,38]. Some authors have found that associations with childhood trauma and anxiety sensitivity are mediated by other mental health symptoms, including trait anxiety and depression [39,40]. However, these studies did not assess childhood medical trauma specifically. It could be that medical trauma, which by definition includes trauma related to one’s body, is more likely to be reciprocally associated with anxiety sensitivity compared to ACEs or traumatic experiences in general. A cross-sectional study by Fair et al. (2022) reported on the high prevalence of anxiety and depressive symptoms in patients with JIA and found correlations with pain and stress [41]. Patients with JIA and other rheumatologic diseases have been found to have more depression and anxiety compared to healthy children, with some evidence to suggest that their psychological comorbidities may impact their pain, disability, and quality of life more than their disease activity [42,43,44]. Efforts to increase engagement in mental healthcare and access to mental health providers in pediatric rheumatology are ongoing [45,46]. The current study further supports the need for improved mental health awareness and screening in this population.

This small study conducted at a single academic center has limitations that should be considered. This was an exploratory cross-sectional study, and no conclusions can be made on the causality of the associations found. Without a larger sample size and longitudinal follow-up, we were unable to assess a wider range of potential confounders (such as geographical location, cultural and racial factors, etc.) or the dynamic nature of disease flares, cumulative pain exposure, or evolving psychological responses over time. The absence of more comprehensive data on disease course variables restricts our ability to fully understand the context of traumatic medical experiences. The lack of a control group limits both the internal validity and the ability to draw condition-specific conclusions about medical PTSD risk. A small number screened positive for likely medical PTSD, further lowering the power to detect smaller associations with medical trauma, in which specific medical experiences may have been traumatic, or to correct for potentially confounding variables. Children below the age of eight years were excluded from this study as the CPSS-V is validated for self-report in ages 8–18 years. This younger population of children with inflammatory arthritis will be important to assess in the future as they may be particularly vulnerable to potentially traumatic medical experiences [47]. Less than a third of our sample had ever been hospitalized, an experience that we had hypothesized could be a source of trauma. Disease activity scores were calculated based on the clinical status at the time of recruitment and do not reflect the severity of each participant’s disease course overall. Our participants had relatively high disease activity scores, which could represent a sampling bias as patients with more severe disease tend to present to the clinic more frequently. Additionally, the majority of ACEs were reported by a parent/guardian, who may not be aware of or wish to acknowledge all ACEs experienced by their child. For those youth who reported medical trauma symptoms related to their arthritis, we did not systematically identify which experiences they found most traumatizing. Finally, formal measurement of psychological symptoms was limited to PTSD and anxiety sensitivity. History of depression and anxiety were measured primarily by parent report only, which is particularly inadequate for older youth who might not share their inner experiences and symptoms with their parents/guardians. This fact limits the ability to quantify current symptom severity and may result in an underestimation of undiagnosed or subclinical distress. These limitations, which are associated with this small exploratory study, point to the need for larger, longitudinal, multicenter studies with more robust symptom measurement, appropriate control groups, and multivariable analyses to clarify the relationships between disease burden, medical trauma, mental health, and pain outcomes in youth with chronic rheumatologic conditions.

To our knowledge, this is the first study to evaluate the prevalence of medical trauma symptoms and explore its association with pain in youth with chronic rheumatologic conditions. Other strengths of this study include the use of self-report measures for the assessment of trauma, self-efficacy, anxiety sensitivity, and pain symptoms in youth that may have been missed if reported solely by parents or guardians.

Future studies including a larger cohort, younger children, and prospective design would help to provide a better understanding of medical trauma, its true prevalence, and the bidirectional nature of the role of pain as it relates to medical trauma in the pediatric rheumatology population. Going forward, further analysis of medical trauma symptoms as a continuous measure may allow for better detection of subthreshold symptoms and associations. Additionally, investigations are needed to examine the impact of provider education on trauma-informed care and the utility of established interventions for medical trauma in pediatric rheumatologic diseases.

Many children and adolescents with inflammatory arthritis reported medical trauma symptoms, with 14% meeting criteria for probable medical PTSD. Medical trauma symptoms were associated with older age, increased pain, anxiety sensitivity, and JIA disease activity. Further research is needed to determine the overall prevalence of medical trauma in pediatric rheumatology patients, its risk factors, impact on functional and clinical outcomes, and potential interventions in this population.

## Figures and Tables

**Table 1:** Demographic characteristics of children and adolescents with chronic inflammatory arthritis, stratified by CPSS-V scores above or below the probable medical PTSD cut-off (≥ 31).

	All Participants (*n* = 50)	CPSS-V < 31 (*n* = 43)	CPSS-V ≥ 31 (*n* = 7)	*p* Value
Age (years), median [IQR] ^1^	15 [13, 17]	15 [12, 17]	17 [16, 17]	0.027
Time since diagnosis (years), median [IQR] ^1^	5 [2, 8]	5 [2, 7]	4 [1.6, 9]	1.000
Sex, n (%) ^2^				0.485
Female	33 (66%)	27 (63%)	6 (86%)	
Male	16 (32%)	15 (35%)	1 (14%)	
No response	1 (2%)	1 (2%)	0 (0%)	
Rheumatologic diagnosis, n (%) ^2^				1.000
JIA	42 (84%)	36 (84%)	6 (86%)	
Oligoarticular	10 (20%)	9 (21%)	1 (14%)	
Polyarticular, RF−	11 (22%)	9 (21%)	2 (29%)	
Polyarticular, RF+	5 (10%)	3 (7%)	2 (29%)	
ERA	9 (18%)	8 (19%)	1 (14%)	
Psoriatic	6 (12%)	6 (14%)	0 (0%)	
Systemic	1 (2%)	1 (2%)	0 (0%)	
cSLE	8 (16%)	7 (16%)	1 (14%)	
Race *, n (%) ^2^				0.611
Armenian	1 (2%)	1 (2%)	0 (0%)	
Asian	8 (16%)	8 (19%)	0 (0%)	
Black or African American	1 (2%)	1 (2%)	0 (0%)	
White	29 (58%)	23 (54%)	6 (86%)	
More than 1 race	4 (8%)	4 (9%)	1 (14%)	
No response	6 (12%)	6 (14%)	0 (0%)	
Ethnicity *, n (%) ^2^				0.403
Hispanic or Latino/a	18 (36%)	15 (35%)	3 (43%)	
Not Hispanic or Latino/a	29 (58%)	26 (61%)	3 (43%)	
No response	3 (6%)	2 (5%)	1 (14%)	
Annual household income *, n (%) ^2^				0.793
<$25,000	1 (2%)	1 (2%)	0 (0%)	
$25–49,999	3 (6%)	2 (5%)	1 (14%)	
$50–99,999	10 (20%)	9 (21%)	1 (14%)	
>$100,000	27 (54%)	23 (54%)	4 (57%)	
No response	9 (18%)	8 (19%)	1 (14%)	
History of *, n (%) ^2^				
Depression	15 (30%)	10 (23%)	5 (71%)	0.020
Anxiety	21 (42%)	16 (37%)	5 (71%)	0.115

Abbreviations: CPSS-V = Child PTSD Symptom Scale for DSM-V; cSLE = childhood-onset systemic lupus erythematosus; ERA = enthesitis-related arthritis; JIA = juvenile idiopathic arthritis; RF = rheumatoid factor. IQR = Interquartile range showing the 25th percentile and 75th percentile. * Self or parent/guardian reported. ^1^ Wilcoxon-Mann-Whitney rank sum test; ^2^ Fisher’s exact test.

**Table 2:** Treatment history and disease activity measures for participants, stratified by CPSS-V scores above or below the probable medical PTSD cut-off (≥31).

	All Participants (*n* = 50)	CPSS-V < 31 (*n* = 43)	CPSS-V ≥ 31 (*n* = 7)	*p* Value
Ever hospitalized, n (%) ^1^	15 (30%)	14 (33%)	1 (14%)	0.659
Ever IV, subQ or IA medications, n (%) ^1^	35 (68%)	30 (70%)	4 (57%)	0.666
^a^ ESR (mm/h), median [IQR] ^2^	8 [3, 21]	7 [3, 18.5]	11 [3, 57]	0.646
^b^ CRP (mg/dL), median [IQR] ^2^	0.3 [0.3, 0.4]	0.3 [0.3, 0.4]	0.3 [0.3, 1.1]	0.642
^c^ cJADAS-10 score, median [IQR] ^2^	8 [2, 11]	7.5 [2, 10.5]	11.5 [9, 14]	0.032
^d^ SLEDAI-2K score, median [IQR] ^2^	8.5 [4, 11]	7 [4, 12]	10	NR

Abbreviations: cJADAS-10 = Clinical Juvenile Arthritis Disease Activity Scores, 10 joints; CPSS-V = Child PTSD Symptom Scale for DSM-V; CRP = C-reactive protein; ESR = erythrocyte sedimentation rate; IA = intraarticular; IV = intravenous; SLEDAI-2K = Systemic Lupus Erythematosus Disease Activity Index 2000; subQ = subcutaneous. IQR = Interquartile range showing the 25th percentile and 75th percentile. NR = Not reportable due to small N. ^a^ N = 39 (32 + 7); ^b^ N = 36 (29 + 7); ^c^ N = 42 (36 + 6); ^d^ N = 8 (6 + 1). ^1^ Fisher’s exact test; ^2^ Wilcoxon-Mann-Whitney rank sum test.

**Table 3:** Questionnaire and tool scores for participants, stratified by CPSS-V scores above or below the probable medical PTSD cut-off (≥31).

	All Participants (*n* = 50)	CPSS-V < 31 (n = 43)	CPSS-V ≥ 31 (*n* = 7)	*p* Value
CPSS-V, median [IQR] ^1^	14 [9, 25]	12 [8, 22]	46 [34, 54]	N/A
ACEs, median [IQR] ^1^	1 [0, 3]	1 [0, 2]	3 [2, 3]	0.005
CSES, median [IQR] ^1^	18.5 [13, 23]	18 [12, 23]	20 [16, 34]	0.149
PSAT				
Met JFM criteria *, n (%) ^2^	9 (18%)	6 (14%)	3 (43%)	0.100
WPI, median [IQR] ^1^	3 [1, 4]	3 [1, 4]	4 [3.5, 7]	0.123
SS, median [IQR] ^1^	5 [3, 7]	5 [3, 7]	8 [6.5, 8.5]	0.039
CASI, median [IQR] ^1^	29.5 [26, 36]	29 [26, 35]	39 [34, 48]	0.008
Pain Score, median [IQR] ^1^	4 [2, 5]	3 [1, 5]	4 [4, 9]	0.043

Abbreviations: ACEs = Adverse Childhood Experiences questionnaire; CASI = Childhood Anxiety Sensitivity Index; CPSS-V = Child PTSD Symptom Scale for DSM-V; CSES = Child Self-Efficacy Sale; N/A = not applicable; PSAT = Pain and Symptom Assessment Tool; SS = Symptom Severity; WPI = Widespread Pain Index. IQR = Interquartile range showing the 25th percentile and 75th percentile. * Defined as WPI ≥ 7 and SS ≥ 5 or WPI 3–6 and SS ≥ 9. ^1^ Wilcoxon-Mann-Whitney rank sum test; ^2^ Fisher’s exact test.

## Data Availability

The raw data supporting the conclusions of this article will be made available by the authors on request due to patient privacy reasons.

## Abbreviations

The following abbreviations are used in this manuscript:

ACEs	Adverse childhood experiences
BARC	Bay Area Research Consortium on Toxic Stress and Health
CARRA	Childhood Arthritis and Rheumatology Research Alliance
CASI	Childhood Anxiety Sensitivity Index
cJADAS	Clinical Juvenile Arthritis Disease Activity Score
CPSS-V	Child PTSD Symptom Scale for DSM-V
CRP	C-reactive protein
CSES	Child Self-Efficacy Scale
cSLE	Childhood-onset systemic lupus erythematous
DMARD	Disease-modifying antirheumatic drug
ERA	Enthesitis-related arthritis
ESR	Erythrocyte sedimentation rate
IQR	Interquartile range
JFM	Juvenile fibromyalgia
JIA	Juvenile idiopathic arthritis
NR	Not reportable
PEARLS	Pediatric ACEs and Related Life-events Screener
PSAT	Post-traumatic stress disorder
RF	Rheumatoid factor
SLEDAI-2K	Systemic Lupus Erythematosus Disease Activity Index 2000
SS	Symptom Severity
WPI	Widespread Pain Index

## References

[1] Sonagra M., Jones J., McGill M., Gmuca S. (2022). Exploring the intersection of adverse childhood experiences, pediatric chronic pain, and rheumatic disease. Pediatr. Rheumatol. Online J..

[2] Groenewald C.B., Murray C.B., Palermo T.M. (2020). Adverse childhood experiences and chronic pain among children and adolescents in the United States. PAIN Rep..

[3] McInnis P.M., Braund T.A., Chua Z.K., Kozlowska K. (2020). Stress-system activation in children with chronic pain: A focus for clinical intervention. Clin. Child Psychol. Psychiatry.

[4] Nelson S., Burns M., McEwen B., Borsook D. (2020). Stressful experiences in youth: “Set-up” for diminished resilience to chronic pain. Brain Behav. Immun.-Health.

[5] Peterson S., Medical Trauma (2018). The National Child Traumatic Stress Network. https://www.nctsn.org/what-is-child-trauma/trauma-types/medical-trauma.

[6] Vinall J., Pavlova M., Asmundson G.J.G., Rasic N., Noel M. (2016). Mental Health Comorbidities in Pediatric Chronic Pain: A Narrative Review of Epidemiology, Models, Neurobiological Mechanisms and Treatment. Children.

[7] Pinquart M. (2020). Posttraumatic Stress Symptoms and Disorders in Children and Adolescents with Chronic Physical Illnesses: A Meta-Analysis. J. Child Adolesc. Trauma.

[8] Kahana S.Y., Feeny N.C., Youngstrom E.A., Drotar D. (2006). Posttraumatic stress in youth experiencing illnesses and injuries: An exploratory meta-analysis. Traumatology.

[9] Price J., Kassam-Adams N., Alderfer M.A., Christofferson J., Kazak A.E. (2016). Systematic Review: A Reevaluation and Update of the Integrative (Trajectory) Model of Pediatric Medical Traumatic Stress. J. Pediatr. Psychol..

[10] Cuneo A.A., Sifflet C., Bardach N., Ly N., von Scheven E., Perito E.R. (2023). Pediatric Medical Traumatic Stress and Trauma-Informed Care in Pediatric Chronic Illness: A Healthcare Provider Survey. J. Pediatr..

[11] Chiu H.T.S., Low D.C.W., Chan A.H.T., Meiser-Stedman R. (2024). Relationship between anxiety sensitivity and post-traumatic stress symptoms in trauma-exposed adults: A meta-analysis. J. Anxiety Disord..

[12] Lies J., Lau S.T., Jones L.E., Jensen M.P., Tan G. (2017). Predictors and Moderators of Post-traumatic Stress Disorder: An Investigation of Anxiety Sensitivity and Resilience in Individuals with Chronic Pain. Ann. Acad. Med. Singap..

[13] Muris P., Vlaeyen J., Meesters C. (2001). The relationship between anxiety sensitivity and fear of pain in healthy adolescents. Behav. Res. Ther..

[14] Beukelman T., Ringold S., Davis T.E., Morgan DeWitt E., Pelajo C.F., Weiss P.F., Kimura Y. (2012). Disease-modifying Antirheumatic Drug Use in the Treatment of Juvenile Idiopathic Arthritis: A Cross-sectional Analysis of the CARRA Registry. J. Rheumatol..

[15] Knight A.M., Weiss P.F., Morales K.H., Keren R. (2014). National Trends in Pediatric Systemic Lupus Erythematosus Hospitalization in the United States: 2000–2009. J. Rheumatol..

[16] Trincianti C., Van Dijkhuizen E.H.P., Alongi A., Mazzoni M., Swart J.F., Nikishina I., Lahdenne P., Rutkowska-Sak L., Avcin T., Quartier P. (2021). Definition and Validation of the American College of Rheumatology 2021 Juvenile Arthritis Disease Activity Score Cutoffs for Disease Activity States in Juvenile Idiopathic Arthritis. Arthritis Rheumatol..

[17] Yee C.-S., Farewell V.T., Isenberg D.A., Griffiths B., Teh L.S., Bruce I.N., Ahmad Y., Rahman A., Prabu A., Akil M. (2011). The use of Systemic Lupus Erythematosus Disease Activity Index-2000 to define active disease and minimal clinically meaningful change based on data from a large cohort of systemic lupus erythematosus patients. Rheumatology.

[18] Foa E.B., Asnaani A., Zang Y., Capaldi S., Yeh R. (2018). Psychometrics of the Child PTSD Symptom Scale for DSM-5 for Trauma-Exposed Children and Adolescents. J. Clin. Child Adolesc. Psychol..

[19] Hosoda-Urban T., O’Donnell E.H. (2024). Diabetes-Related Posttraumatic Stress Symptoms in Adolescents and Young Adults with Type 1 Diabetes: A Pilot Study. J. Acad. Consult.-Liaison Psychiatry.

[20] Taft T.H., Quinton S., Jedel S., Simons M., Mutlu E.A., Hanauer S.B. (2022). Posttraumatic Stress in Patients with Inflammatory Bowel Disease: Prevalence and Relationships to Patient-Reported Outcomes. Inflamm. Bowel Dis..

[21] Bursch B., Tsao J.C.I., Meldrum M., Zeltzer L.K. (2006). Preliminary validation of a self-efficacy scale for child functioning despite chronic pain (child and parent versions). Pain.

[22] Silverman W.K., Fleisig W., Rabian B., Peterson R.A. (1991). Childhood Anxiety Sensitivity Index. J. Clin. Child Psychol..

[23] Koita K., Long D., Hessler D., Benson M., Daley K., Bucci M., Thakur N., Burke Harris N. (2018). Development and implementation of a pediatric adverse childhood experiences (ACEs) and other determinants of health questionnaire in the pediatric medical home: A pilot study. PLoS ONE.

[24] Daffin M., Gibler R.C., Kashikar-Zuck S. (2020). Measures of Juvenile Fibromyalgia: Pain and Symptom Assessment Tool (PSAT), PROMIS^®^ Pain Interference, Anxiety and Depression scales, Functional Disability Inventory (FDI) and Pediatric Quality of Life (PedsQL) 3.0 Rheumatology Module. Arthritis Care Res..

[25] R Core Team (2022). R: A Language and Environment for Statistical Computing. https://www.R-project.org.

[26] Forgey M., Bursch B. (2013). Assessment and management of pediatric iatrogenic medical trauma. Curr. Psychiatry Rep..

[27] Cunningham N.R., Tran S.T., Lynch-Jordan A.M., Ting T.V., Sil S., Strotman D., Noll J.G., Powers S.W., Arnold L.M., Kashikar-Zuck S. (2015). Psychiatric Disorders in Young Adults Diagnosed with Juvenile Fibromyalgia in Adolescence. J. Rheumatol..

[28] Nelson S., Cunningham N., Peugh J., Jagpal A., Arnold L.M., Lynch-Jordan A., Kashikar-Zuck S. (2017). Clinical Profiles of Young Adults with Juvenile-Onset Fibromyalgia with and Without a History of Trauma. Arthritis Care Res..

[29] Goldschen L., Ellrodt J., Amonoo H.L., Feldman C.H., Case S.M., Koenen K.C., Kubzansky L.D., Costenbader K.H. (2023). The link between post-traumatic stress disorder and systemic lupus erythematosus. Brain Behav. Immun..

[30] Case S.M., Feldman C.H., Guan H., Stevens E., Kubzansky L.D., Koenen K.C., Costenbader K.H. (2021). Posttraumatic Stress Disorder and Risk of Systemic Lupus Erythematosus Among Medicaid Recipients. Arthritis Care Res..

[31] Rubinstein T.B., Bullock D.R., Ardalan K., Mowrey W.B., Brown N.M., Bauman L.J., Stein R.E.K. (2020). Adverse Childhood Experiences Are Associated with Childhood-Onset Arthritis in a National Sample of US Youth: An Analysis of the 2016 National Survey of Children’s Health. J. Pediatr..

[32] Patterson S., Trupin L., Hartogensis W., DeQuattro K., Lanata C., Gordon C., Barbour K.E., Greenlund K.J., Dall’Era M., Yazdany J. (2023). Perceived Stress and Prediction of Worse Disease Activity and Symptoms in a Multiracial, Multiethnic Systemic Lupus Erythematosus Cohort. Arthritis Care Res..

[33] Katz P., Patterson S.L., DeQuattro K., Lanata C.M., Barbour K.E., Greenlund K.J., Gordon C., Criswell L.A., Dall’Era M., Yazdany J. (2023). The association of trauma with self-reported flares and disease activity in systemic lupus erythematosus (SLE). Rheumatology.

[34] Marks R. (2014). Self-efficacy and arthritis disability: An updated synthesis of the evidence base and its relevance to optimal patient care. Health Psychol. Open.

[35] Vuorimaa H., Tamm K., Honkanen V., Konttinen Y.T. (2008). Empirical classification of children with JIA: A multidimensional approach to pain and well-being. Clin. Exp. Rheumatol..

[36] Kazak A.E., Kassam-Adams N., Schneider S., Zelikovsky N., Alderfer M.A., Rourke M. (2006). An integrative model of pediatric medical traumatic stress. J. Pediatr. Psychol..

[37] Martin A.L., McGrath P.A., Brown S.C., Katz J. (2007). Anxiety sensitivity, fear of pain and pain-related disability in children and adolescents with chronic pain. Pain Res. Manag..

[38] Mahrer N.E., Montaño Z., Gold J.I. (2012). Relations between anxiety sensitivity, somatization, and health-related quality of life in children with chronic pain. J. Pediatr. Psychol..

[39] Martin L., Viljoen M., Kidd M., Seedat S. (2014). Are childhood trauma exposures predictive of anxiety sensitivity in school attending youth?. J. Affect. Disord..

[40] Delgado-Sanchez A., Brown C., Charalambous C., Sivan M., Jones A. (2023). Trauma in childhood is associated with greater pain catastrophizing but not anxiety sensitivity: A cross-sectional study. Pain Rep..

[41] Fair D.C., Nocton J.J., Panepinto J.A., Yan K., Zhang J., Rodriguez M., Olson J. (2022). Anxiety and Depressive Symptoms in Juvenile Idiopathic Arthritis Correlate with Pain and Stress Using PROMIS Measures. J. Rheumatol..

[42] Fair D.C., Rodriguez M., Knight A.M., Rubinstein T.B. (2019). Depression and Anxiety in Patients with Juvenile Idiopathic Arthritis: Current Insights and Impact on Quality of Life, A Systematic Review. Open Access Rheumatol. Res. Rev..

[43] Davis A.M., Rubinstein T.B., Rodriguez M., Knight A.M. (2017). Mental health care for youth with rheumatologic diseases—Bridging the gap. Pediatr. Rheumatol..

[44] Hanns L., Radziszewska A., Suffield L., Josephs F., Chaplin H., Peckham H., Sen D., Christie D., Carvalho L.A., Ioannou Y. (2020). Association of Anxiety with Pain and Disability but Not with Increased Measures of Inflammation in Adolescent Patients with Juvenile Idiopathic Arthritis. Arthritis Care Res..

[45] Goldstein-Leever A., Bearer C., Sivaraman V., Akoghlanian S., Gallup J., Ardoin S. (2023). Increasing access to psychological services within pediatric rheumatology care. Pediatr. Rheumatol..

[46] Fawole O.A., Reed M.V., Harris J.G., Hersh A., Rodriguez M., Onel K., Lawson E., Rubinstein T., Ardalan K., Morgan E. (2021). Engaging patients and parents to improve mental health intervention for youth with rheumatological disease. Pediatr. Rheumatol..

[47] De Young A.C., Paterson R.S., Brown E.A., Egberts M.R., Le Brocque R.M., Kenardy J.A., Landolt M.A., Marsac M.L., Alisic E., Haag A.C. (2021). Topical Review: Medical Trauma During Early Childhood. J. Pediatr. Psychol..

